# Latent Profiles of Problematic Short‐Video Use and Presenteeism Among Hospital Nurses: The Mediating Role of Work Interference With Personal Life

**DOI:** 10.1002/nop2.70879

**Published:** 2026-10-01

**Authors:** Zhaoping Liu, Mengsi Li, Yang Liu, Pan Yang, Wen Liu, Bei Hu

**Affiliations:** ^1^ Department of Respiratory Medicine General Hospital of Central Theater Command of People's Liberation Army Wuhan Hubei China; ^2^ Department of Anesthesiology, the Affiliated Changsha Central Hospital, Hengyang Medical School University of South China Changsha Changsha Hunan China; ^3^ School of Nursing Hunan Normal University Changsha Hunan China; ^4^ Nursing Department General Hospital of Central Theater Command of People's Liberation Army Wuhan Hubei China

**Keywords:** latent profile analysis, nurses, presenteeism, problematic short‐video use, work interference with personal life

## Abstract

**Aim:**

To identify latent profiles of problematic short‐video use among nurses from three tertiary hospitals in Wuhan, examine factors associated with presenteeism and assess indirect associations through work interference with personal life.

**Design:**

A cross‐sectional survey.

**Methods:**

From October to December 2025, 680 nurses recruited through convenience sampling completed measures of problematic short‐video use, work interference with personal life and presenteeism. Latent profile, hierarchical multiple linear regression and bootstrap path analyses were conducted. The path model adjusted for age, monthly income and night‐shift frequency.

**Results:**

Four profiles were identified: low‐symptom use, moderate escapism–tolerance, high escapism–persistent use and generalized high symptoms. Monthly income, work interference with personal life and membership in the moderate escapism–tolerance and generalized high‐symptom profiles were independently associated with presenteeism. Compared with the low‐symptom profile, all three other profiles were indirectly associated with greater presenteeism through work interference with personal life. Direct associations remained evident for the moderate escapism–tolerance and generalized high‐symptom profiles, but not for the high escapism–persistent‐use profile.

**Conclusion:**

Problematic short‐video use was heterogeneous and associated with nurses' work–nonwork experiences and presenteeism. Work interference with personal life may represent a statistical pathway linking profile membership to presenteeism, although the cross‐sectional design precludes causal or temporal conclusions.

**Implications for the Profession:**

Strategies that protect off‐job recovery, support healthy digital boundaries and reduce work interference with personal life warrant evaluation.

**Impact:**

This study identifies distinct problematic‐use profiles and profile‐specific associations with work interference and presenteeism among nurses from three tertiary hospitals.

**Reporting Method:**

The study followed the Strengthening the Reporting of Observational Studies in Epidemiology statement.

**No Patient or Public Contribution:**

Patients and the public were not involved in the study design, conduct, analysis, interpretation or manuscript preparation.

**Abbreviations**
aBIC: sample‐size‐adjusted Bayesian information criterion
AIC: Akaike information criterion
AUC: area under the curve
BIC: Bayesian information criterion
BLRT: bootstrap likelihood ratio test
CFI: comparative fit index
CI: confidence interval
df: degrees of freedom
ICC: intraclass correlation coefficient
IP: Internet Protocol
LMRT: Lo–Mendell–Rubin adjusted likelihood ratio test
LPA: latent profile analysis
PSVUS‐9: 9‐item Problematic Short‐Video Use Scale
RMB: renminbi
RMSEA: root mean square error of approximation
ROC: receiver operating characteristic
SD: standard deviation
SE: standard error
SPS‐6: 6‐item Stanford Presenteeism Scale
SPSS: Statistical Package for the Social Sciences
SRMR: standardized root mean square residual
STROBE: Strengthening the Reporting of Observational Studies in Epidemiology
TLI: Tucker–Lewis index
VIF: variance inflation factor
WIPL: work interference with personal life

## 1 Introduction

Short‐video platforms have become a prominent digital media environment in contemporary society. According to the China Internet Network Information Center, the number of short‐video users in China reached 1.05 billion by June 2024, accounting for 95.5% of the country's internet users (China Internet Network Information Center 2024). Under conditions of frequent exposure and algorithm‐driven engagement, some individuals may develop persistent and poorly controlled patterns of use that continue despite adverse consequences and functional impairment, collectively referred to as problematic short‐video use (Ruijuan et al. 2025). Problematic short‐video use may manifest as salience, withdrawal, tolerance, unsuccessful attempts to reduce use, escapism, deception, loss of interest, continued use despite harm and functional impairment (Ruijuan et al. 2025). It has also been associated with poorer sleep quality, procrastination and depressive symptoms (Liao 2024; Zhao and Kou 2024). However, neither short videos nor the smartphones through which they are accessed are inherently harmful. For nurses, mobile devices support information retrieval, team communication, clinical decision‐making and health education. The concern arises when instrumental or recreational use shifts toward uncontrolled, withdrawal‐driven or escapist use that is difficult to discontinue despite its adverse consequences. Previous studies have shown that nurses' personal smartphone use may have both benefits and risks, whereas persistent digital connectivity outside working hours may interfere with psychological detachment and increase work interference with personal life (Blake et al. 2024; de Jong et al. 2020). Therefore, the consequences of short‐video use depend not merely on whether it occurs, but on whether it becomes dysregulated, displaces opportunities for recovery and produces functional impairment within a specific occupational context. In this study, problematic short‐video use was conceptualized not as an occupational demand in itself, but as a behaviour that may displace off‐job recovery opportunities when it becomes uncontrolled, withdrawal‐driven or escapist. Such use during nonwork time may displace sleep, relaxation, psychological detachment and other restorative activities, while also consuming attention and self‐regulatory capacity.

## 2 Background

According to the National Health Commission of the People's Republic of China, the number of registered nurses in China reached 5.63 million by the end of 2023 (National Health Commission of the People's Republic of China 2024). Nurses are routinely exposed to shift schedules, night work, high emotional demands and substantial clinical responsibility, all of which may consume psychological and physical resources and increase their vulnerability to work interference with personal life and presenteeism. Presenteeism generally refers to attending work despite a physical or psychological condition that would otherwise warrant rest or absence (Gerlach et al. 2024). As an important concern in occupational nursing health, presenteeism may worsen nurses' health, reduce their ability to complete work effectively, compromise quality of care and increase patient safety risks. Available evidence suggests that the prevalence of presenteeism among nurses ranges from approximately 32% to 94%, with a substantial burden also reported among Chinese nurses (Wang and Li 2026; Xu et al. 2024). Studies of shift‐working intensive care and emergency nurses have reported positive associations between work‐related interference with family or personal life and presenteeism, suggesting that intrusion of work demands into nonwork life may be accompanied by poorer work functioning (Gen et al. 2024; Li et al. 2024; Wu et al. 2024).

Work interference with personal life refers to the extent to which work demands intrude upon or adversely affect an individual's personal life (Keeney et al. 2013). It represents the unidirectional influence of work on personal life and is conceptually distinct from interference originating in personal life and affecting work. Among clinical nurses, demanding schedules, night shifts, emotional labour and high clinical responsibility consume substantial resources and may cause work demands to spill over into personal life. When nonwork time does not provide sufficient sleep, relaxation and psychological detachment, the resources expended at work may not be adequately replenished, causing work‐related demands to remain cognitively and emotionally salient during personal time. Problematic short‐video use may be relevant to this process because uncontrolled use during nonwork time may reduce the time and self‐regulatory resources available for recovery and personal responsibilities. Rather than increasing objective work demands, problematic use may leave nurses with fewer resources for recovery and boundary management, which may make existing work demands feel more intrusive during personal time. Persistent work interference with personal life may then prolong work‐related cognitive and emotional activation, further restrict recovery and leave nurses fatigued or unable to function fully during subsequent shifts. When nurses remain at work under these conditions, reduced concentration, energy and work capacity may manifest as presenteeism.

According to Conservation of Resources theory, individuals strive to obtain, preserve and protect valued resources, including time, energy, emotional stability and opportunities for recovery. When these resources are threatened, depleted or inadequately replenished, individuals become more vulnerable to stress and functional impairment (Hobfoll 1989). Among clinical nurses, high work intensity, shift and night work, and emotional labour create a context of sustained resource expenditure and a corresponding need for effective off‐job recovery. In this context, when short‐video use becomes escapist, withdrawal‐driven, persistent or functionally impairing, it may compete with restorative activities for limited nonwork time and attentional resources. The proposed mechanism is therefore a recovery opportunity cost: problematic use may displace sleep, relaxation, psychological detachment and other activities needed to replenish resources expended at work. With fewer resources available for recovery and boundary management, nurses may experience existing work demands as intruding more strongly into personal life; work interference with personal life may therefore reflect the difficulty of containing work‐related demands within the work domain when off‐job recovery is insufficient. This interference may further restrict resource replenishment and contribute to fatigue, impaired concentration and reduced work capacity, thereby increasing presenteeism. Conservation of Resources theory therefore supports an indirect pathway linking problematic short‐video use profiles to presenteeism through reduced recovery opportunities and greater work interference with personal life. Nevertheless, because these variables were measured cross‐sectionally, the proposed sequence should be interpreted as a theory‐guided indirect association rather than a confirmed causal mechanism. Reverse or reciprocal relationships also remain possible; for example, occupational stress or work interference with personal life may encourage escapist short‐video use.

Existing nursing research on digital media use has primarily focused on smartphone addiction, work procrastination and burnout, whereas evidence concerning problematic short‐video use among clinical nurses remains limited (Ma et al. 2021; Xue et al. 2024). Few studies have examined problematic short‐video use, work interference with personal life and presenteeism within the same analytical framework. Moreover, previous studies have predominantly relied on total scores, which describe overall severity but may obscure heterogeneity in the configuration of problematic‐use symptoms. A person‐centred approach may identify subgroups characterized by different combinations of escapism, withdrawal, persistent use and functional impairment, thereby providing a more nuanced basis for risk identification. Guided by conservation of resources theory, this study used latent profile analysis to identify problematic short‐video use profiles among nurses recruited from three tertiary hospitals in Wuhan, Hubei, China and examined whether profile membership was indirectly associated with presenteeism through work interference with personal life. We hypothesized that: (H1) profiles with more pronounced problematic‐use symptoms would show greater work interference with personal life and higher presenteeism; (H2) work interference with personal life would be positively associated with presenteeism and (H3) profile membership would be indirectly associated with presenteeism through work interference with personal life (Figure 1).

**FIGURE 1:**
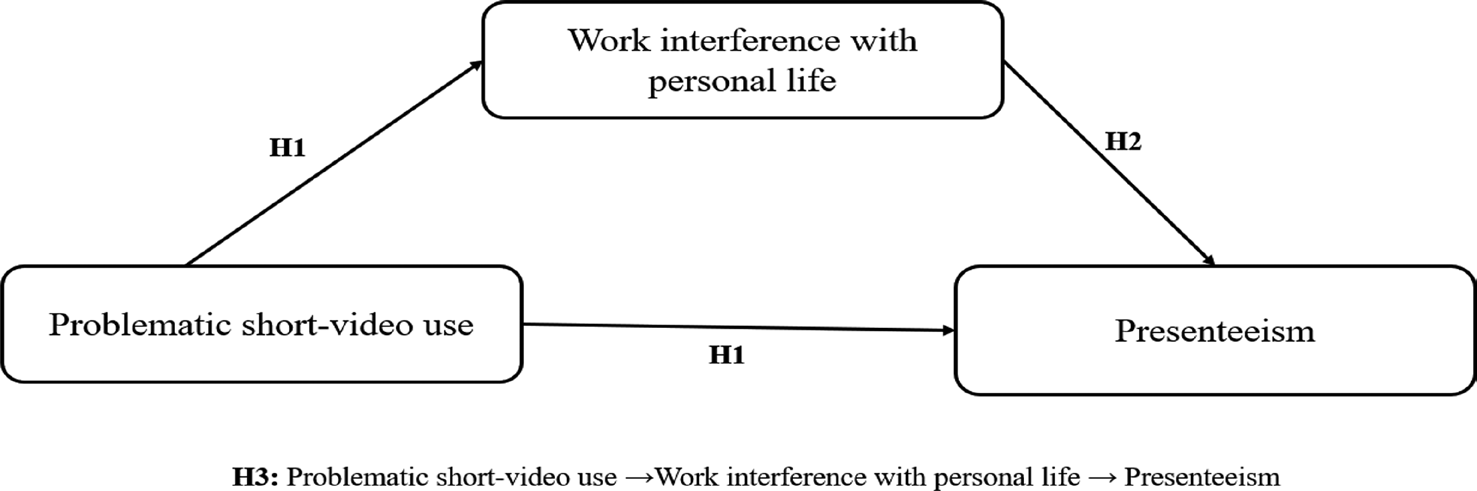
Hypothesized mediation model of problematic short‐video use profiles and presenteeism.

## 3 Aims of the Study

This study aimed to: (1) identify distinct profiles of problematic short‐video use among nurses recruited from three tertiary hospitals in Wuhan, China; (2) identify sociodemographic and occupational factors associated with presenteeism and examine the independent association between problematic short‐video use profiles and presenteeism and (3) examine whether profile membership was indirectly associated with presenteeism through work interference with personal life.

## 4 Methods

### 4.1 Study Design

This cross‐sectional study was reported in accordance with the STROBE statement (Von Elm et al. 2007).

### 4.2 Setting and Sample

Between October and December 2025, clinical nurses from three tertiary hospitals in Wuhan were recruited using convenience sampling. Participants were eligible if they (1) held a valid nursing licence, (2) were aged 18 years or older and (3) had been working in their current post for at least 6 months. Nurses undertaking external training or internships and those unavailable during data collection because of statutory leave or illness were excluded.

Because LPA sample‐size requirements depend on class separation, the number of indicators and classes, class proportions and model‐selection criteria, no single closed‐form formula was applied (Nylund et al. 2007; Tein et al. 2013). Based on previous simulation studies evaluating sample sizes of 200–1000 across models of varying complexity, a conservative target of at least 500 valid responses was adopted for the planned analysis of nine indicators. For the mediation analysis, Fritz and MacKinnon (2007) indicated that 558 participants were required to achieve 80% power using percentile‐bootstrap testing when both component paths had small standardized coefficients of 0.14. The larger requirement was therefore selected. Allowing for a 20% invalid‐response rate, the minimum recruitment target was 698, which was rounded to 700 nurses.

### 4.3 Variables and Instruments

#### 4.3.1 Sociodemographic Information

The questionnaire was developed by the researchers based on a review of the relevant literature and consultation within the research team, and covered gender, age, marital status, educational attainment, years of employment, professional title, department, monthly income and monthly number of night shifts.

#### 4.3.2 Problematic Short‐Video Use Scale‐9 (PSVUS‐9)

Problematic short‐video use was measured using the 9‐item Problematic Short Video Use Scale (PSVUS‐9), developed and validated by Ruijuan et al. 2025. The scale comprises nine items assessing nine symptoms of problematic short‐video use: salience, withdrawal, tolerance, failed attempts to reduce use, escapism, deception, loss of interest, functional impairment and continued use despite harm. Items were rated on a 6‐point Likert scale ranging from 0 (‘never’) to 5 (‘always’). Total scores were obtained by summing the item scores, with higher scores indicating more severe problematic short‐video use. Following the original validation study, a total score of ≥ 24 was used as a screening threshold for elevated problematic short‐video use. At the corresponding ROC cutoff of 23.5, the original study reported an AUC of 0.990, sensitivity of 0.953 and specificity of 0.948 using the LPA‐derived classification; using clinician classification, the AUC, sensitivity and specificity were 0.894, 0.857 and 0.753, respectively. The threshold was selected for screening sensitivity and was not considered diagnostic. The original PSVUS‐9 showed acceptable structural validity (*χ* ^2^/df = 3.27, CFI = 0.96, TLI = 0.94, RMSEA = 0.07 and SRMR = 0.04), internal consistency (Cronbach's *α* = 0.86) and 2‐week test–retest reliability (ICC = 0.80). Cronbach's *α* was 0.907 in the present sample.

#### 4.3.3 Stanford Presenteeism Scale (SPS‐6)

Presenteeism was measured using the Stanford Presenteeism Scale (SPS‐6), developed by Koopman et al. (2002) and translated and adapted into Chinese by Zhao et al. (2010). This scale assesses respondents' tendency toward presenteeism during the previous month. The scale includes six items that assess two dimensions: effects on the work process and effects on work outcomes. Items are rated on a 5‐point Likert scale, and items 5 and 6 are reverse coded. The total score ranges from 6 to 30, and higher scores indicate greater levels of presenteeism. In this study, the scale demonstrated a Cronbach's *α* of 0.965.

#### 4.3.4 Work–Interference With Personal Life Scale

Work interference with personal life was assessed using the corresponding five‐item subscale of the Work/Nonwork Interference and Enhancement Scale developed by Fisher et al. (2009). The present study used only the validated five‐item work–interference‐with‐personal‐life subscale because the hypothesized pathway concerned the unidirectional intrusion of work into personal life. The subscale includes five items (e.g., ‘My personal life is affected by my work’), each rated on a 5‐point Likert scale. The psychometric properties of this subscale, including its reliability and validity, have been adequately established in the study by Hirschi et al. (2019). In this study, the subscale demonstrated a Cronbach's *α* of 0.943.

### 4.4 Data Collection

The online questionnaire was developed using Wenjuanxing (https://www.wjx.cn/), a specialized online survey platform based in China. The questionnaire was disseminated through WeChat, a widely used social communication application in China. The leader of the research team contacted the nursing departments of the participating hospitals and obtained permission to conduct the survey. Following approval, the questionnaire link was distributed through the WeChat groups used by nurses, allowing participants to access and complete the questionnaire using their mobile devices.

All items were mandatory to prevent incomplete submissions. The survey platform restricted repeated submissions from the same WeChat account, device or IP address and recorded completion time for quality assessment. Submitted questionnaires were screened for duplicate submissions, logical inconsistencies and invariant response patterns. Of the 700 questionnaires, 20 unique questionnaires were excluded: 12 showed predefined logical inconsistencies, including two duplicate submissions completed in less than 60 s and eight showed invariant responding. Completion time alone was not used as the sole exclusion criterion. Questionnaires meeting more than one exclusion criterion were counted only once. The remaining 680 questionnaires passed all data‐quality checks and contained no item‐level missing data. Among the included questionnaires, the median completion time was 311.50 s, with an interquartile range of 220.00–474.75 s. The data were stored anonymously and were accessible only to members of the research team.

### 4.5 Data Analysis

Analyses were conducted using SPSS 27.0 and Mplus 8.3. Descriptive statistics summarized participant characteristics. Presenteeism scores were compared using independent‐samples *t*‐tests or one‐way analysis of variance, and continuous variables were examined using Pearson correlations. One‐ to five‐class latent profile models were estimated. Model selection jointly considered AIC, BIC, aBIC, LMRT, BLRT, entropy, class size, profile distinctiveness and theoretical interpretability. Entropy was interpreted as an index of overall classification certainty, not individual classification accuracy. Hierarchical multiple linear regression was used to identify factors independently associated with presenteeism; categorical variables were dummy‐coded and multicollinearity was assessed using variance inflation factors. An observed‐variable path model examined indirect associations between profile membership and presenteeism through work interference with personal life. Indirect effects were evaluated using 5000 bootstrap samples and bias‐corrected 95% confidence intervals. All tests were two‐sided with *α* = 0.05. Because no item‐level data were missing, complete‐case analysis was used without imputation.

## 5 Results

### 5.1 Participant Characteristics and Univariate Analysis of Presenteeism

A total of 680 nurses were included in the analysis. Most participants were female (94.0%), aged 34 years or younger (59.5%), married (68.8%) and educated to the bachelor's degree level (81.3%). Regarding occupational characteristics, 58.7% had worked in nursing for 6–15 years, 48.8% held an intermediate professional title and the largest proportions worked in internal medicine (34.4%) or surgical departments (21.9%). Approximately half of the participants reported a monthly income of RMB 5000–10,000 (51.2%), and 64.1% worked at least four evening or night shifts per month. Univariate analyses showed that presenteeism scores differed according to gender, age, years of nursing employment, professional title, department, monthly income and evening/night‐shift frequency (all *p* < 0.05), but not according to marital status or educational level (both *p* > 0.05; Table 1).

**TABLE 1:** Participant characteristics and univariate analysis of presenteeism among nurses recruited from three tertiary hospitals.

Characteristic	Category	*N*	Mean ± SD	*t*/*F*	*p*
Gender				4.269	0.039
	Male	41	17.268 ± 5.710		
	Female	639	15.172 ± 6.332		
Age (years)				4.863	0.002
	< 30	200	15.995 ± 6.442		
	30–34	205	14.849 ± 6.236		
	35–39	178	16.045 ± 6.464		
	≥ 40	97	13.443 ± 5.489		
Marital status				1.066	0.302
	Unmarried/divorced/widowed	212	15.670 ± 6.091		
	Married	468	15.130 ± 6.409		
Educational level				0.325	0.723
	Associate degree or below	93	15.290 ± 6.798		
	Bachelor's degree	553	15.248 ± 6.236		
	Master's degree or above	34	16.147 ± 6.306		
Years in practice (years)				4.793	0.003
	≤ 5	157	15.905 ± 6.361		
	6–10	199	15.578 ± 6.458		
	11–15	200	15.730 ± 6.337		
	> 15	124	13.387 ± 5.652		
Professional title				5.567	0.004
	Junior	322	15.351 ± 6.381		
	Intermediate	332	15.560 ± 6.226		
	Associate senior and above	26	11.308 ± 5.342		
Department				2.652	0.022
	Internal medicine	234	14.748 ± 5.920		
	Surgery	149	16.517 ± 6.323		
	Intensive care unit	63	16.460 ± 6.567		
	Emergency department	63	13.873 ± 6.052		
	Obstetrics/gynaecology and paediatrics	35	15.543 ± 7.318		
	Other departments	136	14.971 ± 6.488		
Monthly income (RMB)				4.081	0.017
	< 5000	230	16.222 ± 6.541		
	5000–10,000	348	14.957 ± 6.318		
	> 10,000	102	14.382 ± 5.524		
Average number of night shifts per month				4.884	0.002
	None	121	14.273 ± 6.163		
	≤ 3	123	14.312 ± 6.234		
	4–6	229	15.231 ± 6.203		
	≥ 7	207	16.556 ± 6.388		

*Note:* Independent‐samples *t*‐tests or one‐way analyses of variance were used, as appropriate. A *p*‐value < 0.05 was considered statistically significant. ‘None’ indicates that the participant reported no evening or night shifts during an average month.

Abbreviation: SD, standard deviation.

### 5.2 Descriptive Statistics and Correlations Among the Study Variables

The mean scores for problematic short‐video use, work interference with personal life and presenteeism were 21.23 ± 8.80, 16.17 ± 5.39 and 15.30 ± 6.13, respectively. Overall, 38.4% of participants met the PSVUS‐9 screening threshold of ≥ 24. Problematic short‐video use was positively correlated with work interference with personal life (*r* = 0.305) and presenteeism (*r* = 0.370), while work interference with personal life was strongly correlated with presenteeism (*r* = 0.668; all *p* < 0.001; Table 2).

**TABLE 2:** Descriptive statistics and correlation heatmap for the study variables.

Variable	Mean ± SD	PSVUS‐9	WIPL	SPS‐6
PSVUS‐9	21.23 ± 8.80	**1.000**	0.305	0.370
WIPL	16.17 ± 5.39	0.305	**1.000**	0.668
SPS‐6	15.30 ± 6.13	0.370	0.668	**1.000**

*Note:* Values in the correlation matrix are Pearson correlation coefficients. Darker shading indicates a stronger positive correlation. All correlations were statistically significant at *p*‐value < 0.001.

Abbreviations: PSVUS‐9, 9‐item Problematic Short‐Video Use Scale; SD, standard deviation; SPS‐6, 6‐item Stanford Presenteeism Scale; WIPL, work interference with personal life.

### 5.3 Latent Profile Analysis of Problematic Short‐Video Use

One‐ to five‐class latent profile models were estimated sequentially, with fit indices presented in Table 3. AIC, BIC and aBIC decreased as the number of classes increased. Compared with the three‐class solution, the four‐class model had lower information criteria, significant LMRT and BLRT results, high entropy (0.904) and no class containing fewer than 5% of participants. Although the five‐class model had lower information criteria, its LMRT was not significant (*p* = 0.588) and its entropy decreased to 0.873. The four‐class solution was therefore retained as the best balance of relative fit, classification certainty, class size, profile distinctiveness and theoretical interpretability. The four‐class solution had an entropy of 0.904, indicating high overall classification certainty; entropy was interpreted as an index of overall class separation rather than an individual probability of correct classification.

**TABLE 3:** Fit indices for one‐ to five‐class latent profile models of problematic short‐video use.

Model	AIC	BIC	aBIC	Entropy	LMRT *p*‐value	BLRT *p*‐value	Class proportion
1	20346.21	20427.61	20370.45				
2	17953.75	18080.37	17991.47	0.929	< 0.001	< 0.001	0.616/0.384
3	17263.60	17435.44	17314.79	0.928	0.001	< 0.001	0.563/0.332/0.105
4	17014.67	17231.73	17079.32	0.904	0.033	< 0.001	0.509/0.303/0.090/0.098
5	16757.01	17019.29	16835.14	0.873	0.588	< 0.001	0.326/0.357/0.108/0.085/0.124

*Note:* A *p*‐value < 0.05 for the LMRT or BLRT indicates that the k‐class model provides a significantly better fit than the *k* − 1‐class model. Entropy reflects overall classification certainty, with values closer to 1 indicating clearer class separation. Blank cells indicate that the statistic was not applicable to the one‐class model.

Abbreviations: aBIC, sample‐size‐adjusted Bayesian information criterion; AIC, Akaike information criterion; BIC, Bayesian information criterion; BLRT, bootstrap likelihood ratio test; LMRT, Lo–Mendell–Rubin adjusted likelihood ratio test.

The retained model identified four profiles: C1, the low‐symptom use profile (50.9%); C2, the moderate escapism–tolerance profile (30.3%); C3, the high escapism–persistent‐use profile (9.0%) and C4, the generalized high‐symptom profile (9.8%). C1 showed consistently low scores across all nine symptoms and was therefore labelled the low‐symptom use profile; C2 showed moderate elevations across several symptoms, particularly tolerance, failed attempts to reduce use and escapism, and was labelled the moderate escapism–tolerance profile; C3 showed marked elevations in tolerance, failed attempts to reduce use, escapism and continued use despite harm, but comparatively lower withdrawal, deception and functional impairment; it was therefore labelled the high escapism–persistent‐use profile; C4 showed consistently high scores across nearly all symptoms and was labelled the generalized high‐symptom profile (Figure 2).

**FIGURE 2:**
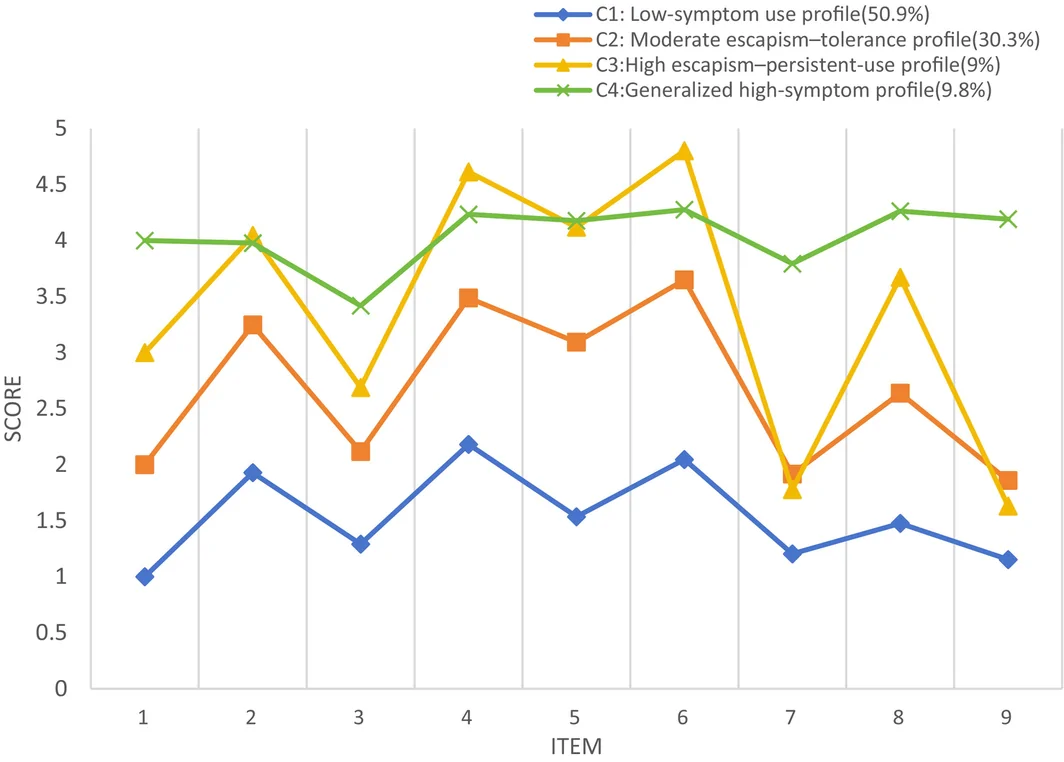
Item‐response means across the four latent profiles of problematic short‐video use.

### 5.4 Hierarchical Multiple Linear Regression Analysis of Presenteeism

Sociodemographic and occupational characteristics explained 9.2% of the variance in presenteeism, with work interference with personal life and problematic short‐video use profiles contributing an additional 38.2% and 3.2%, respectively; the final model explained 50.6% of the variance (adjusted *R* ^2^ = 0.489; all F tests, *p* < 0.001). In the final model, monthly income was the only sociodemographic or occupational characteristic associated with presenteeism at the *p* < 0.05 level. Compared with participants earning < RMB 5000 per month, those earning RMB 5000–10,000 (*β* = −0.077, *p* = 0.016) and > RMB 10,000 (*β* = −0.079, *p* = 0.014) had lower presenteeism scores. Work interference with personal life was positively associated with presenteeism and had the largest standardized coefficient (*β* = 0.601, *p* < 0.001). With C1 as the reference profile, C2 (*β* = 0.122, *p* < 0.001) and C4 (*β* = 0.176, *p* < 0.001) were associated with higher presenteeism scores, whereas C3 was not (*β* = 0.030, *p* = 0.313). VIF values ranged from 1.071 to 6.570 (Table 4).

**TABLE 4:** Hierarchical multiple linear regression analysis of presenteeism: model summary and final‐model coefficients.

Variable	*B*	SE	*β*	*t*	*p*	VIF
Constant	6.663	1.666	—	3.999	< 0.001	—
Gender						
Male	−1.111	0.752	−0.042	−1.477	0.140	1.071
Female (Ref)						
Age (years)						
30–34	−0.680	0.646	−0.049	−1.053	0.293	2.935
35–39	0.582	0.836	0.041	0.697	0.486	4.507
≥ 40	0.346	1.199	0.019	0.288	0.773	5.873
< 30 (Ref)						
Years in practice (years)						
6–10	−0.092	0.619	−0.007	−0.149	0.882	2.648
11–15	−0.612	0.818	−0.044	−0.748	0.455	4.641
> 15	−2.049	1.148	−0.125	−1.784	0.075	6.570
≤ 5 (Ref)						
Professional title						
Intermediate	0.742	0.502	0.059	1.478	0.140	2.105
Associate senior and above	−0.588	1.107	−0.018	−0.531	0.595	1.506
Junior (Ref)						
Monthly income (RMB)						
5000–10,000	−0.970	0.403	−0.077	−2.408	0.016	1.355
> 10,000	−1.395	0.568	−0.079	−2.458	0.014	1.372
< 5000 (Ref)						
Average number of night shifts per month						
≤ 3	−1.189	0.605	−0.073	−1.964	0.050	1.814
4–6	−0.836	0.553	−0.063	−1.512	0.131	2.279
≥ 7	−0.125	0.571	−0.009	−0.219	0.826	2.306
None (Ref)						
Department						
Surgery	0.410	0.487	0.027	0.841	0.401	1.357
Intensive care unit	0.362	0.658	0.017	0.550	0.582	1.218
Emergency	−0.685	0.662	−0.032	−1.035	0.301	1.232
Obstetrics, gynaecology and paediatrics	−0.630	0.840	−0.022	−0.750	0.453	1.150
Other	−0.358	0.505	−0.023	−0.708	0.479	1.365
Internal medicine (Ref)						
Problematic short‐video use profile						
C2	1.672	0.408	0.122	4.102	< 0.001	1.172
C3	0.661	0.654	0.030	1.011	0.313	1.168
C4	3.725	0.629	0.176	5.922	< 0.001	1.174
C1 (Ref)						
Work interference with personal life	0.704	0.035	0.601	20.257	< 0.001	1.171

*Note:* Model 1 included sociodemographic and occupational variables and explained 9.2% of the variance in presenteeism (adjusted *R* ^2^ = 0.066). Model 2 added work interference with personal life, accounting for an additional 38.2% of the variance (adjusted *R* ^2^ = 0.458). Model 3 further added problematic short‐video use profile membership, explaining an additional 3.2% of the variance; the final model explained 50.6% of the total variance in presenteeism (adjusted *R* ^2^ = 0.489; all *F*‐change tests, *p*‐value < 0.001). ‘—’ indicates that the statistic was not applicable.

Abbreviations: *β*, standardized coefficient; *B*, unstandardized coefficient; Ref, reference category; SE, standard error; VIF, variance inflation factor.

### 5.5 Indirect Associations Through Work Interference With Personal Life

The observed‐variable path model was saturated (*χ* ^2^ = 0, df = 0, CFI = 1.000, TLI = 1.000, RMSEA = 0 and SRMR = 0). Because these indices are mathematically determined in a saturated model and do not independently indicate model adequacy, interpretation focused on the path coefficients, bias‐corrected bootstrap confidence intervals and explained variance. With C1 as the reference profile, an observed‐variable path model was fitted with adjustment for age, monthly income and monthly evening/night‐shift frequency, which were selected by integrating theoretical relevance with the hierarchical regression findings. Compared with C1, C2 (*B* = 1.555, 95% CI 0.682–2.399), C3 (*B* = 4.184, 95% CI 2.960–5.391) and C4 (*B* = 3.997, 95% CI 2.847–5.159) were associated with greater work interference with personal life (all *p* < 0.001). Work interference with personal life was positively associated with presenteeism (*B* = 0.718, 95% CI 0.645–0.788, *p* < 0.001). After accounting for work interference with personal life and the covariates, direct associations with presenteeism remained significant for C2 (*B* = 1.752, 95% CI 0.969–2.529, *p* < 0.001) and C4 (*B* = 3.817, 95% CI 2.507–5.082, *p* < 0.001), but not for C3 (*B* = 0.675, 95% CI −0.857 to 2.081, *p* = 0.364). Bias‐corrected bootstrap analyses showed significant indirect associations through work interference with personal life for C2 (*B* = 1.117, 95% CI 0.492–1.768, *p* = 0.001), C3 (*B* = 3.004, 95% CI 2.107–3.960, *p* < 0.001) and C4 (*B* = 2.870, 95% CI 2.016–3.783, *p* < 0.001). The corresponding total associations were 2.868 (95% CI 1.893–3.793), 3.679 (95% CI 2.022–5.309) and 6.687 (95% CI 5.061–8.220), respectively (all *p* < 0.001). The model explained 11.5% of the variance in work interference with personal life and 49.5% of the variance in presenteeism (Table 5 and Figure 3).

**TABLE 5:** Direct, indirect and total associations between problematic short‐video use profiles and presenteeism through work interference with personal life.

Path	*B*	SE	*β*	Bootstrap 95% CI	*p*
Profile → WIPL (*a* paths)					
C2 → WIPL	1.555	0.441	0.133	0.682–2.399	< 0.001
C3 → WIPL	4.184	0.615	0.222	2.960–5.391	< 0.001
C4 → WIPL	3.997	0.598	0.221	2.847–5.159	< 0.001
WIPL → presenteeism (*b* path)					
WIPL → presenteeism	0.718	0.036	0.613	0.645–0.788	< 0.001
Direct associations with presenteeism (*c*′ paths)					
C2 → presenteeism	1.752	0.398	0.128	0.969–2.529	< 0.001
C3 → presenteeism	0.675	0.743	0.031	−0.857‐2.081	0.364
C4 → presenteeism	3.817	0.659	0.18	2.507–5.082	< 0.001
Indirect associations through WIPL (*a* × *b*)					
C2	1.117	0.324	—	0.492–1.768	0.001
C3	3.004	0.471	—	2.107–3.960	< 0.001
C4	2.87	0.453	—	2.016–3.783	< 0.001
Total associations (*c*)					
C2	2.868	0.492	—	1.893–3.793	< 0.001
C3	3.679	0.83	—	2.022–5.309	< 0.001
C4	6.687	0.818	—	5.061–8.220	< 0.001

*Note:* C1, the low‐symptom use profile, was the reference group. ‘—’ indicates that a standardized coefficient was not estimated for the indirect or total association. Statistical significance was determined when the bias‐corrected bootstrap 95% CI did not include zero.

Abbreviations: *β*, standardized coefficient; *B*, unstandardized coefficient; CI, confidence interval; SE, standard error; WIPL, work interference with personal life.

**FIGURE 3:**
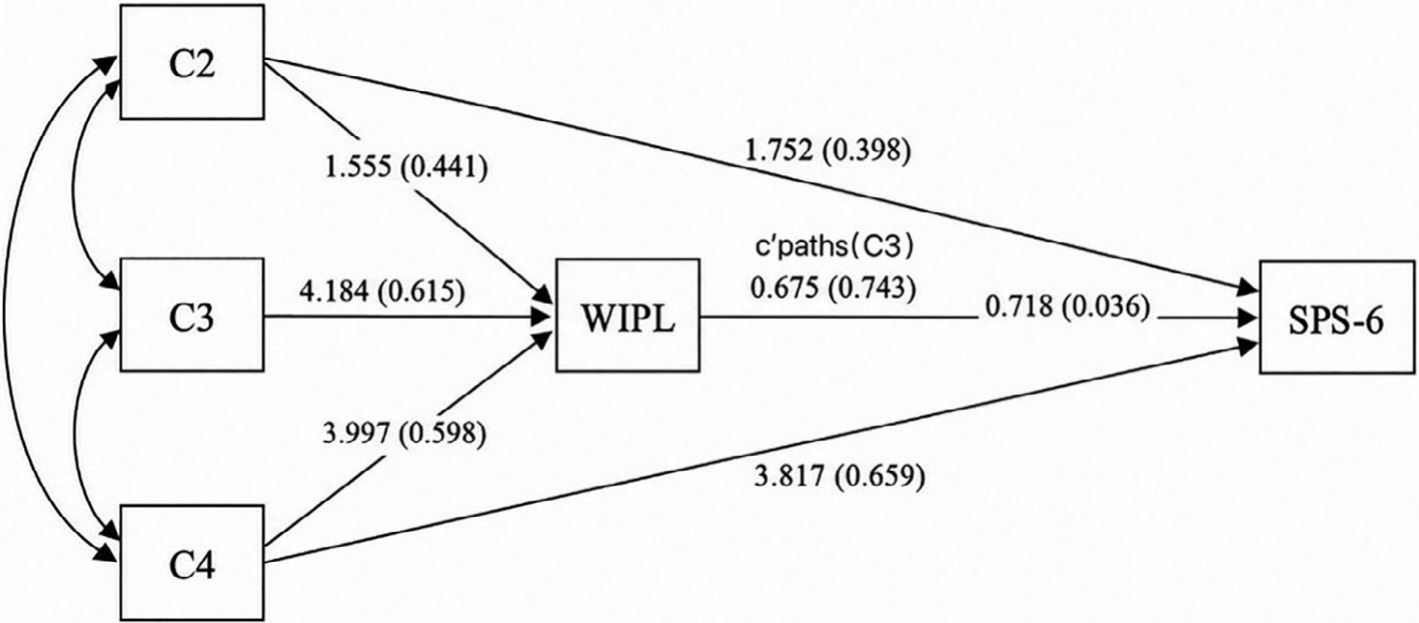
Mediation pathway of work interference with personal life in the association between problematic short‐video use and presenteeism.

## 6 Discussion

In this study, 38.4% of nurses recruited from three tertiary hospitals in Wuhan met the PSVUS‐9 screening threshold for elevated problematic short‐video use. Problematic short‐video use, work interference with personal life and presenteeism were positively correlated, indicating that these phenomena tended to co‐occur within the study sample. The findings extend previous research on digital‐media use by showing that symptom configurations, rather than total severity alone, may be relevant to nurses' work–nonwork experiences and work functioning (Aldhafeeri et al. 2025; Ruijuan et al. 2025; Simsekli et al. 2025; Yan 2024). Taken together, the findings are consistent with, but do not directly demonstrate, a recovery‐based explanation linking problematic short‐video use, work interference with personal life and presenteeism.

### 6.1 Heterogeneity in Problematic Short‐Video Use Profiles

Using the nine PSVUS‐9 items, this study identified four profiles: low‐symptom use, moderate escapism–tolerance, high escapism–persistent use and generalized high symptoms. This four‐profile solution is consistent with research identifying graded short‐video addiction profiles among Chinese college students (Liu et al. 2025). However, previous studies have identified three to five profiles, suggesting that profile structures may vary by population, instrument, indicators and model‐selection criteria (Chen et al. 2026; Jiang et al. 2025). The generalized high‐symptom profile accounted for 9.8% of the sample, similar to the high‐risk short‐video addiction profile reported by Liu et al. (10.89%) and the problematic social media use profile reported by Peng et al. (9.8%). These proportions are not directly comparable because the studies used different samples and measures. The moderate escapism–tolerance profile showed increasing involvement and impaired control without generalized functional impairment. This finding supports evidence that tolerance or high engagement alone may not indicate problematic use (Jiang et al. 2025; Peng and Liao 2023). In contrast, the high escapism–persistent‐use profile showed pronounced escapism, tolerance, failed control and continued use despite harm, but relatively low deception and functional impairment. This uneven pattern extends previous severity‐based classifications by suggesting qualitative differences in symptom expression. The generalized high‐symptom profile showed elevated scores across nearly all symptoms and therefore represented the broadest symptom burden. These findings suggest that individuals with similar overall levels of problematic short‐video use may differ in their dominant symptom patterns. Nevertheless, the profiles represent relative response patterns within this sample and should not be interpreted as validated diagnostic or clinical risk categories.

### 6.2 Factors Associated With Presenteeism

Univariate analyses showed differences in presenteeism across several sociodemographic and occupational groups; however, most of these differences were not retained after simultaneous adjustment. In the final hierarchical regression model, monthly income was the only sociodemographic or occupational characteristic associated with presenteeism at the *p* < 0.05 level. Participants in the two higher‐income groups reported lower presenteeism scores than those earning < RMB 5000 per month. Previous research among Chinese nurses has identified financial needs, workload, leave arrangements and professional responsibility as possible reasons for working while unwell (Shan et al. 2020). The present income finding may therefore reflect differences in financial pressure or employment circumstances, although these mechanisms were not directly measured. Work interference with personal life accounted for the largest increase in explained variance and had the largest standardized coefficient in the final model. This is consistent with evidence linking work‐to‐family conflict and impaired work–life balance to poorer work functioning and presenteeism among nurses (Amstad et al. 2011; Rainbow et al. 2021; Zhang et al. 2023). Profile membership explained a further 3.2% of the variance after the other variables were entered, suggesting that symptom configuration provided modest additional information beyond sociodemographic characteristics and work interference with personal life.

### 6.3 Work Interference With Personal Life as an Indirect Explanatory Pathway

Compared with C1, C2–C4 were indirectly associated with higher presenteeism through greater work interference with personal life. Direct associations remained significant for C2 and C4 but not C3. This supports an indirect statistical pathway rather than causal mediation. Previous nursing studies have similarly linked work–family conflict with presenteeism, although the present study specifically measured the unidirectional interference of work with personal life (Zhang et al. 2025, 2023). Conservation of Resources theory offers a possible explanation. Problematic short‐video use during nonwork time may displace sleep, relaxation and psychological detachment, thereby reducing opportunities to restore resources expended at work. Inadequate recovery may increase perceived work intrusion into personal life and subsequently be accompanied by reduced energy, concentration or work capacity. For C3, the significant indirect but nonsignificant direct association suggests that its relationship with presenteeism was statistically concentrated in this pathway, but does not prove complete mediation. The remaining direct associations for C2 and C4 imply additional unmeasured mechanisms, such as sleep disruption or emotional exhaustion. Reverse or cyclical relationships are also plausible because work interference or presenteeism may increase escapist short‐video use, potentially creating a resource‐loss cycle (Demerouti et al. 2009).

### 6.4 Implications for Nursing Management

These profiles may help identify different patterns of problematic short‐video use but should not be treated as validated screening or diagnostic categories. Universal education may help nurses resembling C1 maintain healthy digital boundaries and protect recovery time. Support for C2 or C3 may emphasize identifying escapist or uncontrolled use, alternative emotion‐regulation strategies and device‐free periods before sleep. Nurses resembling C4 may benefit from confidential assessment of sleep, emotional exhaustion, recovery and work functioning, with referral when impairment persists. Individual strategies may be insufficient when organizational demands remain high. Evidence links job demands to greater work–family conflict and work–family‐specific organizational support to lower conflict (French et al. 2018; Ghislieri et al. 2017; Kossek et al. 2011). Practical measures include predictable scheduling, adequate intershift rest, flexible shift exchange, protected breaks, supervisor training and confidential occupational‐health services. These measures should be supportive rather than punitive because restricting personal digital‐media use alone does not address occupational stress or inadequate recovery.

### 6.5 Strengths, Limitations and Future Research

This study integrated problematic short‐video use profiles, work interference with personal life and presenteeism within a person‐centred framework, capturing symptom heterogeneity and profile‐specific direct and indirect associations.

Several limitations should be considered. First, the cross‐sectional design precluded conclusions about temporal order, causal mediation or reverse relationships. Second, convenience sampling from three tertiary hospitals in Wuhan may have introduced selection bias and limited generalizability to nurses in other regions and healthcare settings. The small number of male participants also reduced the precision of sex‐specific comparisons. Third, reliance on self‐reported data may have introduced recall, social‐desirability and common‐method biases. Fourth, only work interference with personal life was measured; the findings cannot be generalized to the reverse direction or bidirectional work–life conflict. Fifth, the proposed recovery mechanism remains theoretical because sleep, psychological detachment and emotional exhaustion were not directly measured. Finally, the profiles require external validation, and analyses based on most‐likely class membership may not fully account for classification uncertainty.

Future studies should validate the four‐profile solution in multi‐centre samples and use longitudinal designs to clarify temporal and reciprocal relationships. Incorporating objective short‐video use, recovery indicators and organizational support would enable more direct testing of the proposed mechanisms.

## 7 Conclusion

This study identified four profiles of problematic short‐video use among nurses from three tertiary hospitals in Wuhan: low‐symptom use, moderate escapism–tolerance, high escapism–persistent use and generalized high symptoms. Compared with the low‐symptom profile, the other profiles were indirectly associated with higher presenteeism through greater work interference with personal life, although direct associations varied across profiles. These exploratory associations do not establish causality or diagnostic categories and require validation in multicenter longitudinal studies with more diverse nursing samples.

## Author Contributions

**Zhaoping Liu:** conceptualization, methodology, data curation, writing – review and editing, writing – original draft, investigation, formal analysis. **Mengsi Li:** conceptualization, methodology, data curation, investigation, writing – original draft, writing – review and editing. **Yang Liu:** formal analysis, data curation, methodology, writing – review and editing. **Pan Yang:** investigation, formal analysis, writing – review and editing. **Wen Liu:** conceptualization, methodology, software, writing – review and editing, project administration, supervision. **Bei Hu:** conceptualization, methodology, formal analysis, validation, investigation, writing – review and editing, supervision.

## Funding

The authors have nothing to report.

## Ethics Statement

The study has received ethical approval from the Ethics Committee of Hunan Normal University, China (Application No. 2025(844), Date: 11/10/2025). Respondent were explicitly informed in the study that they were under no obligation to participate or complete the questionnaire. Additionally, participants were assured that the questionnaire was anonymous and that all data would be kept strictly confidential.

## Conflicts of Interest

The authors declare no conflicts of interest.

## Data Availability

The data that support the findings of this study are available from the corresponding author upon reasonable request.
